# *OsRACK1A*, encodes a circadian clock-regulated WD40 protein, negatively affect salt tolerance in rice

**DOI:** 10.1186/s12284-018-0232-3

**Published:** 2018-08-02

**Authors:** Dongping Zhang, Yuzhu Wang, Jinyu Shen, Jianfeng Yin, Dahong Li, Yan Gao, Weifeng Xu, Jiansheng Liang

**Affiliations:** 1 Jiangsu Key Laboratory of Crop Genetics and Physiology/Co-Innovation Center for Modern Production Technology of Grain Crop, Yangzhou University, Yangzhou, 225009 Jiangsu China; 2 College of Life Sciences, Fujian Agriculture and Forestry University, Jinshan, Fuzhou, 350002 China; 3 Department of Biological Engineering, Huanghuai University, Zhumadian, 463000 Henan China; 4 Department of Biology, Southern University of Science and Technology, Shenzhen, 518055 China

**Keywords:** OsRACK1A, Salt tolerance, Circadian, Rice

## Abstract

The receptor for activated C kinase 1 (RACK1) is a WD40 type protein that is involved in multiple signaling pathways and is conserved from prokaryotes to eukaryotes. Here we report that rice *RACK1A* (*OsRACK1A*) is regulated by circadian clocks and plays an important role in the salt stress response. *OsRACK1A* was found to follow a rhythmic expression profile under circadian conditions at both the transcription and the translation levels, although the expression was arrhythmic under salt stress. Analysis of plant survival rates, fresh weight, proline content, malondialdehyde, and chlorophyll showed that suppression of *OsRACK1A* enhanced tolerance to salt stress. The ion concentration in both roots and leaves revealed that *OsRACK1A*-suppressed transgenic rice could maintain low Na^+^ and high K^+^ concentrations. Furthermore, *OsRACK1A*-suppressed transgenic rice accumulated significantly more abscisic acid (ABA) and more transcripts of ABA- and stress-inducible genes compared with the wild-type plants. Real-time quantitative polymerase chain reaction analysis revealed that many stress-related genes, including APETALA 2/Ethylene Responsive Factor (AP2/ERF) transcription factors, were upregulated in the *OsRACK1A*-suppressed transgenic rice line. We identified putative interactors of OsRACK1A, and found that OsRACK1A interacted with many salt stress-responsive proteins directly. These results suggest that *OsRACK1A* is regulated by circadian rhythm, and involved in the regulation of salt stress responses.

**Electronic supplementary material:**

The online version of this article (10.1186/s12284-018-0232-3) contains supplementary material, which is available to authorized users.

## Background

The receptor for activated C kinase 1 (RACK1) is a member of the WD repeat-containing scaffold proteins and is conserved from prokaryotes to eukaryotes (Zhang et al., 2013). As a scaffolding protein, RACK1 protein interacts with many proteins and is involved in multiple signaling pathways (McCahill et al., 2002; Zhang et al., 2013). In plants, *RACK1* is involved in diverse biological processes, such as seed germination, organ development, hormones and stress responses (Nakashima et al., 2008; Guo et al., 2009, 2011; Zhang et al., 2014). Compared with the advances made from studies in metazoans and yeast, little is known about the molecular mechanisms of *RACK1* in plants.

The *Arabidopsis* genome contains three *RACK1* orthologues, *RACK1A*, *RACK1B* and *RACK1C*, which are ~ 78% similar to mammalian *RACK1* (Guo and Chen, 2008). Using loss-of-function mutants of *RACK1A* in *Arabidopsis*, Chen et al.(2006) found that *AtRACK1A* plays a role in several plant hormonal responses, including abscisic acid (ABA), gibberellin (GA), indole-3-acetic acid (IAA), and brassinosteroid (BR). There is direct and indirect evidence that *RACK1s* are involved in the regulation of plant tolerance to abiotic and biotic stresses (Kundu et al., 2013; Cheng et al., 2015). In *Arabidopsis*, the *rack1a* mutant strongly tolerates soil drying, compared with the wild-type (Zhang et al., 2013). Moreover, water loss in detached leaves and stomatal conductance of *rack1* mutants were significantly lower than in the wild-type, and the endogenous ABA content of *rack1a* mutants was higher than in the wild-type (Guo et al., 2009; Zhang et al., 2013). In addition, *rack1a* mutants were hypersensitive to ABA in serval developmental processes, such as seed germination, cotyledon greening, and root growth, and some ABA-responsive marker genes were upregulated in *rack1a* mutants, while the *RACK1* genes were downregulated by ABA (Guo et al., 2009). These results suggest that *RACK1* functions as a negative regulator of ABA signaling and consequently enhances drought stress tolerance via ABA-dependent signaling in response to water stress in plants. Comparative proteomic analysis showed that the *Arabidopsis* RACK1C protein might play roles in regulating plant resistance to salt stress (Shi et al., 2011).

The rice genome contains two *RACK1* homologous genes that are ~ 80% similar to *Arabidopsis* RACK1 proteins at the amino acid level: *OsRACK1A* and *OsRACK1B* (Nakashima et al., 2008). Li et al. (2009) found that *OsRACK1A*-suppressed transgenic rice lines were more tolerant of soil drying, but the molecular mechanism remains unknown. Comparative phosphoproteomics studies revealed that the OsRACK1A protein is phosphorylated in response to exogenous ABA and drought treatment (He et al., 2008; Ke et al., 2009). These findings suggested that *OsRACK1A* plays essential roles in ABA signaling and is involved in ABA-dependent stress responses. In addition to the involvement of *RACK1* in the regulation of plant responses to abiotic stresses, it has been reported to function in plant innate immunity. Overexpression of *OsRACK1A* enhanced the production of reactive oxygen species (ROS) and increased resistance to blast fungus in rice (Nakashima et al., 2008). *OsRACK1A* regulated ROS levels not only in abiotic stress responses but also in the seed germination process. Previously, we found that *OsRACK1A* positively regulated seed germination by promoting H_2_O_2_ production and enhancing ABA catabolism (Zhang et al., 2014). Although *RACK1* functions in ABA signaling in both rice and *Arabidopsis*, it is still unclear whether *RACK1* is involved directly in ABA-dependent stress responses.

Circadian clocks are 24-h biological oscillators, which generally enable organisms to coordinate their activities with the external light/dark cycles by anticipating the onset of dawn or dusk. In mammals, RACK1 protein plays a crucial role in circadian clocks by interacting with BMAL1, a component of the heterodimeric CLOCK:BMAL1 circadian complex. However, the expression of *RACK1* itself showed little or no circadian variation across the circadian cycle (Robles et al., 2010). In plants, no clock component has been reported to interact with RACK1 protein and whether plant *RACK1* is involved directly in circadian clock regulation has yet to be investigated. In this study, our results indicated that *OsRACK1A* is a circadian rhythm gene and is involved in the response to salt stress. *OsRACK1A*-suppressed transgenic plants were hyposensitive to salt stress, compared with wild-type Nipponbare. OsRACK1A plays an important role in the tolerance to high salinity by regulating many stress-related genes and interacting directly with many stress-response proteins.

## Methods

### Plant materials and stress treatment

Rice (*Oryza sativa* L. cv. Nipponbare) was used as the wild-type (non-transgenic line; NTL) and in the generation of all transgenic plants. All transgenic rice lines were generated and kept in our laboratory. An *OsRACK1A* over-expressing transgenic line, OeTL3–8, and an RNA-interfered transgenic line, RiTL4–2, were used as experimental materials (Zhang et al., 2014). For NaCl treatment, 4-week-old hydroponic cultured rice plants were placed in different concentrations of NaCl solution (100, 150, 200 mM) for 10–20 d and finally determined 150 mM NaCl treated with 18 d and recovered for 10 d was the best condition for identifying stress phenotypes. All the plants grew in a plant growth chamber (Conviron atc26, 16 h light/ 8 h dark, 30 °C day/ 22 °C night).

### Measurements o**f** physiological index

For the tolerance experiments, all rice plants were cultured in a plant growth chamber (Conviron atc26) (30 °C day / 22 °C night). The survival rate and fresh weight were calculated after 18 d of treatment with 150 mM NaCl and recovery in normal conditions for another 10 d. Lipid peroxidation was determined by measuring the MDA content (Dhindsa and Matowe, 1981). The content of free proline in leaves was determined as described previously. (Bates et al., 1973) Chlorophyll was extracted from the leaves in 10 mL of 80% acetone for 16 h in the dark and was determined by measuring the absorbance at 652 nm (Arnon, 1949). To measure the Na^+^ and K^+^ concentrations, 2-week-old hydroponic cultured rice seedlings were supplemented with 150 mM NaCl for 24, 48, or 72 h. Shoots and roots were harvested at the indicated times and all physiological measurements were based on the procedure described by Yang et al. (2015). Measurement of water loss form detached leaves was performed as previously described by Zhang et al. (2015). The detached leaves of non-transgenic and transgenic rice lines were weighed at room temperature (~ 23 °C) with 35% relative humidity. The endogenous ABA levels of rice leaves were measured based on the procedures described by Zhang et al. (2014). All of the data were subjected to Student’s t-test analysis using SPSS ver. 13.0 (SPSS Company, Chicago, IL).

### Gene expression analysis

The RNAprep Pure Plant Kit (cat. no. DP441; Tiangen Biotech) was used to extract total RNA from rice. Single-strand cDNAs were synthesized by using the HiScript Q RT SuperMix for qPCR kit (cat. no. R123; Vazyme). Transcript-level expression of each gene were measured by quantitative RT-PCR using a 7300 Real-Time PCR system (ABI), with the iTaq universal SYBR Green SuperMix (Bio-Rad), and normalized against the values obtained for housekeeping gene *OsActin1* (LOC_Os03g50890). Three biological replicates were performed for each experiment. Additional file 1: Table S2 lists the qRT-PCR primer sequences. All of the data were subjected to Student’s t-test analysis using SPSS ver. 13.0 (SPSS Company, Chicago, IL).

### Protein blot analysis

Rice leaves of seedlings were ground in liquid nitrogen and homogenized in PBS buffer (cat. no. CW0040S; CoWin Bioscience) containing complete protease inhibitor cocktail (cat. no. 04693132001; Roche). To prepare total protein, the homogenate was centrifuged (6000×*g*, 30 min, 4 °C) to remove cellular debris. Then, proteins were separated by sodium dodecyl sulfate polyacrylamide gel electrophoresis (SDS-PAGE) on 10% gels and blotted onto polyvinylidene difluoride (PVDF) membranes. The antibodies used were anti-β-actin antibody (cat. no. CW0264M; CoWin Bioscience), anti-green fluorescence protein (GFP) antibody (cat. no. ab290; Abcam), and anti-OsRACK1A antibody (cat. no. AbP80112-A-SE; Beijing Protein Innovation).

### Co-IP assay

To identify the OsRACK1A interaction proteins, *UBI::GFP* and *UBI::GFP-OsRACK1A* transgenic rice were used for co-IP assays. Leaves from 4-week-old plants were harvested and ground in liquid nitrogen. Proteins were extracted with the buffer containing 50 mM Tris (pH 7.5), 150 mM NaCl, 0.1% IGEPAL CA-630, Proteinase Inhibitor Cocktail (cat. no. 04693159001; Roche) and Phophatase Inhibitor Cocktail (cat. no. 04906845001). The samples were centrifuged at 12,000 g for 15 min at 4 °C and the supernatant was incubated with anti-GFP magnetic beads (catalog no. D153–11; MBL) to overnight at 4 °C with gentle rotation. The beads were then washed four times with PBS. The immunoprecipitated proteins were eluted with 1 M glycine (pH 3.0). The presence of the corresponding proteins was detected by tandem liquid chromatograph-mass spectrometry (LC-MS/MS).

### Statistical analyses

Statistical analyses were performed using IBM SPSS Statistics 21 software (Chicago, IL, USA), and analyzed with one-way ANOVA. *P* < 0.05 was considered significant.

## Results

### Expression of the *OsRACK1A* gene is controlled in a circadian-clock like manner

**Fig. 1:**
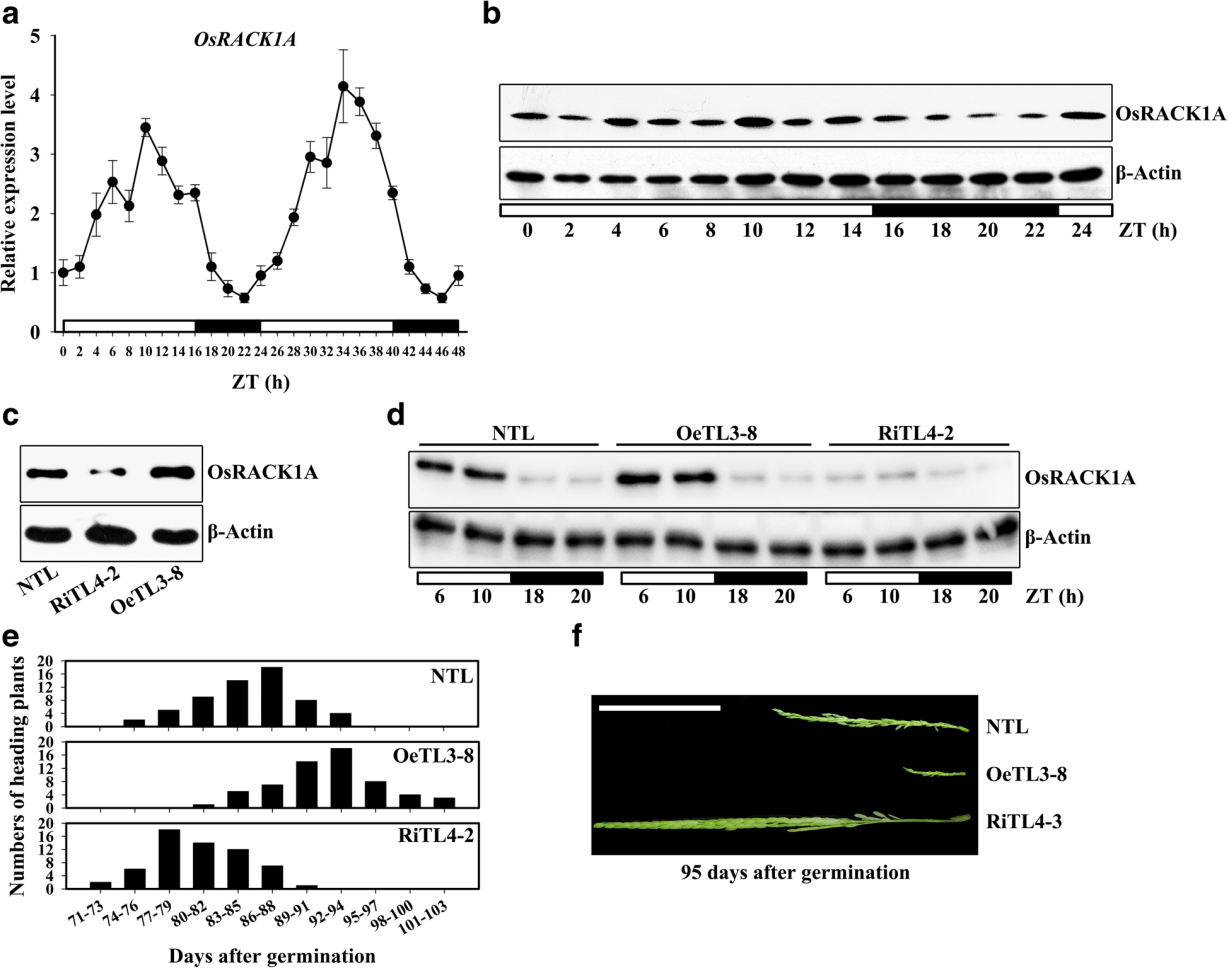
Circadian clock-controlled *OsRACK1A* expression in rice. **a** Transcript analysis of the *OsRACK1A* gene by qRT-PCR over a 48-h period. Rice plants were grown under 16 h light and 8 h dark in a growth chamber at a constant temperature of 28 °C and sampled at regular intervals. **b** Immunoblot analysis of OsRACK1A protein over a 24-h period. **c** *OsRACK1A* expression was monitored by immunoblot analysis in seedlings of a non-transgenic line (NTL), an *OsRACK1A*-overexpressing transgenic line (OeTL3–8), and an RNA-interfered transgenic line (RiTLs4–2). **d** Protein levels of OsRACK1A in NTL, OeTL3–8 and RiTLs4–2 in light and dark. **e** Heading time distributions in transgenic rice plants grown in the field. During the heading period, the number of heading plants for each line was recorded every 3 days and compared. **f** Phenotypic comparison panicles during the heading period of transgenic plants. The rice plants had been grown in the field for 89 d. ZT, Zeitgeber Time. White and black rectangles indicate lights on and lights off, respectively

Information retrieved from the public microarray database (ArrayExpress, Accession: E-MTAB-275) showed that a circadian rhythm in *OsRACK1A* mRNA abundance occurred under photocycling (12 h light/12 h dark; 12 h hot/12 h hot; LDHH), thermocycling (12 h light/12 h light; 12 h hot/ 12 h cold; LLHC) or photocycling and thermocycling (12 h light/12 h dark; 12 h hot/12 h cold; LDHC) conditions (Additional file 1: Figure S1A). Another rice *RACK1* homolog, *OsRACK1B*, exhibited similar expression patterns (Additional file 1: Figure S1B). To confirm whether the expression of *OsRACK1A* was controlled by a circadian clock, the expression of *OsRACK1A* in a 24-h period was measured. Quantitative RT-PCR analysis showed that the transcript level of *OsRACK1A* started accumulating with the onset of light and reached a maximum level 10 h after the lights were switched on (ZT0 and ZT24) and then the transcript level declined gradually and reached a minimum 6 h after the lights were switched off (ZT16 and ZT40, Fig. 1a). We also examined levels of OsRACK1A protein during the light/dark cycle using western blot analysis and the results revealed that OsRACK1A protein accumulated in the light (ZT0 to ZT14) and declined in the dark (ZT16 to ZT22, Fig. 1b). Moreover, we tested the expression of *OsRACK1A* under the constant light conditions and found that *OsRACK1A* also displayed rhythmic expression (Additional file 1: Figure S1C).

### Overexpression of *OsRACK1A* delays the time of heading

Some circadian clock-controlled genes have been reported to be involved in photoperiodic flowering regulation (Xue et al., 2008; Ishikawa et al., 2011; Matsubara et al., 2011). To investigate whether *OsRACK1A* plays a role in photoperiod-controlled heading, we generated several *OsRACK1A* RNA-interference (RNAi) and overexpressing lines (Li et al., 2009). From these transgenic rice lines, we chose the stable downregulated RNA-interfered transgenic line RiTL4–2, and the upregulated overexpressed transgenic line OeTL3–8. Compared with the non-transgenic line (NTL), the OsRACK1A protein level was higher in OeTL3–8 and lower in RiTL4–2, measured by Western blot analysis using an OsRACK1A-specific antibody (Fig. 1c). This differential protein expression patterns of the three genotypes was occurred only in the light, whereas OsRACK1A protein was nearly undetectable in the dark (Fig. 1d). The heading date of field-grown plants was recorded and OeTL3–8 had a heading date nearly 1 week later than NTL, whereas RiTL4–2 showed a heading time approximately 1 week earlier than NTL (Fig. 1e). The panicles phenotypes of field-grown plants at 95 d after germination are shown in Fig. 1f.

### NaCl treatment affects expression of the *OsRACK1A* gene

**Fig. 2:**
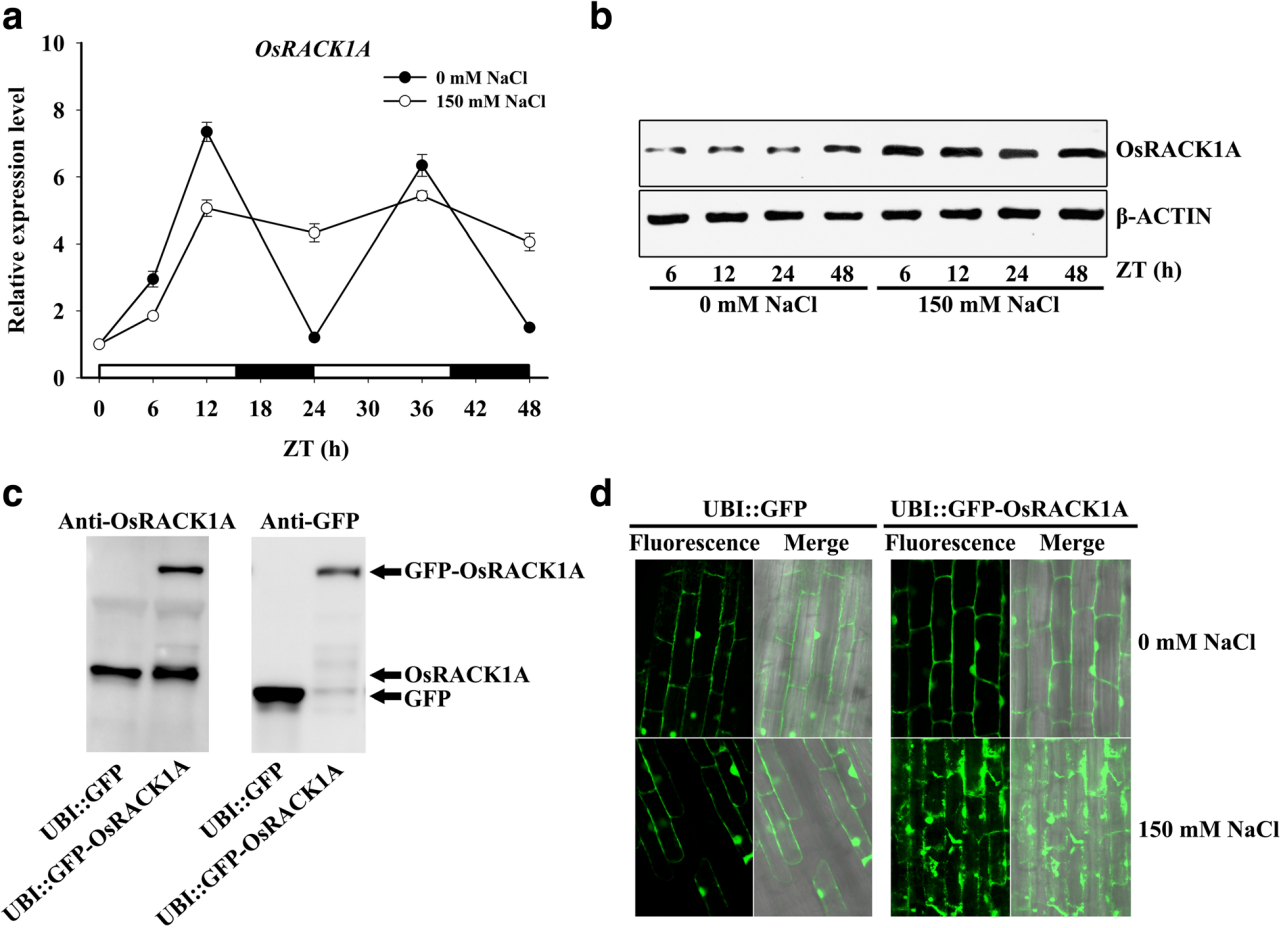
Salt stress-controlled *OsRACK1A* expression in rice. **a** Transcript analysis of the *OsRACK1A* gene by qRT-PCR for a 48-h period under 0 and 150 mM NaCl treatment. **b** Immunoblot analysis of OsRACK1A protein for a 24-h period under 0 and 150 mM NaCl treatment. **c** GFP, OsRACK1A and a GFP-OsRACK1A fusion protein were monitored by immunoblot analysis in seedlings of *UBI*-promoted *GFP* and *GFP-OsRACK1A* transgenic rice. **d** GFP fluorescence was examined in roots of *UBI*-promoted *GFP* and *GFP-OsRACK1A* transgenic rice under 0 and 150 mM NaCl treatment for 6 h. ZT, Zeitgeber Time. White and black rectangles indicate lights on and lights off, respectively

To investigate the expression profile of the *OsRACK1A* gene under salt stress, 2-week-old hydroponic cultured rice seedlings were exposed to 150 mM NaCl for different times and the transcript-level expression of this gene was monitored using quantitative RT-PCR. The *OsRACK1A* expression pattern changed significantly in response to NaCl treatment. The transcript level of *OsRACK1A* accumulated from ZT0 to ZT12 under both salt stress and control conditions, but expression of *OsRACK1A* was slightly higher under control than under salt stress conditions (Fig. 2a). Under normal conditions, the expression of *OsRACK1A* behaved like a circadian clock; when treated with NaCl, however, the transcription level of *OsRACK1A* first increased to a relatively high level and then maintained this level throughout the experiment (Fig. 2a), which means that the circadian clock of *OsRACK1A* expression disappeared when exposed to salt stress. The similar expression pattern of *OsRACK1A* was shown under both light/dark cycle and constant light conditions with NaCl treatment (Additional file 1: Figure S1B). Interestingly, the protein level of OsRACK1A increased and was significantly higher than in the untreated control at 6, 12, 24, and 48 h after the onset of salt stress (Fig. 2b), whereas the transcript levels of *OsRACK1A* were lower at ZT6 and ZT12 in the NaCl treatment than under control conditions (Fig. 2a).

Therefore, we suspected that OsRACK1A protein levels were under post transcriptional and/or translational control. To test this, transgenic rice plants were generated that constitutively expressed GFP-OsRACK1A. As shown in Fig. 2c, the GFP-OsRACK1A fusion protein was detected in ubiquitin-promoted *GFP-OsRACK1A* transgenic plants, whereas the GFP protein was detected in *UBI::GFP* transgenic plants. Figure 2d shows fluorescence images of *GFP-OsRACK1A* transgenic plants in the presence or absence of 150 mM NaCl for 6 h. Control plants containing GFP alone showed no change in subcellular localization in response to salt stress (Fig. 2d). Furthermore, before salt treatment, plants containing GFP-OsRACK1A exhibited fluorescence that was detectable in the cytosolic fraction, as well as in the plasma membrane and nuclei. After treatment with 150 mM NaCl, GFP fluorescence from the GFP-OsRACK1A fusion was enhanced and appeared diffusely in the cytosol (Fig. 2d). These results supported the premise that OsRACK1A protein was controlled by post-transcriptional and/or translational regulation and accumulated under salt stress.

### *OsRACK1A* negatively regulates salt tolerance

**Fig. 3:**
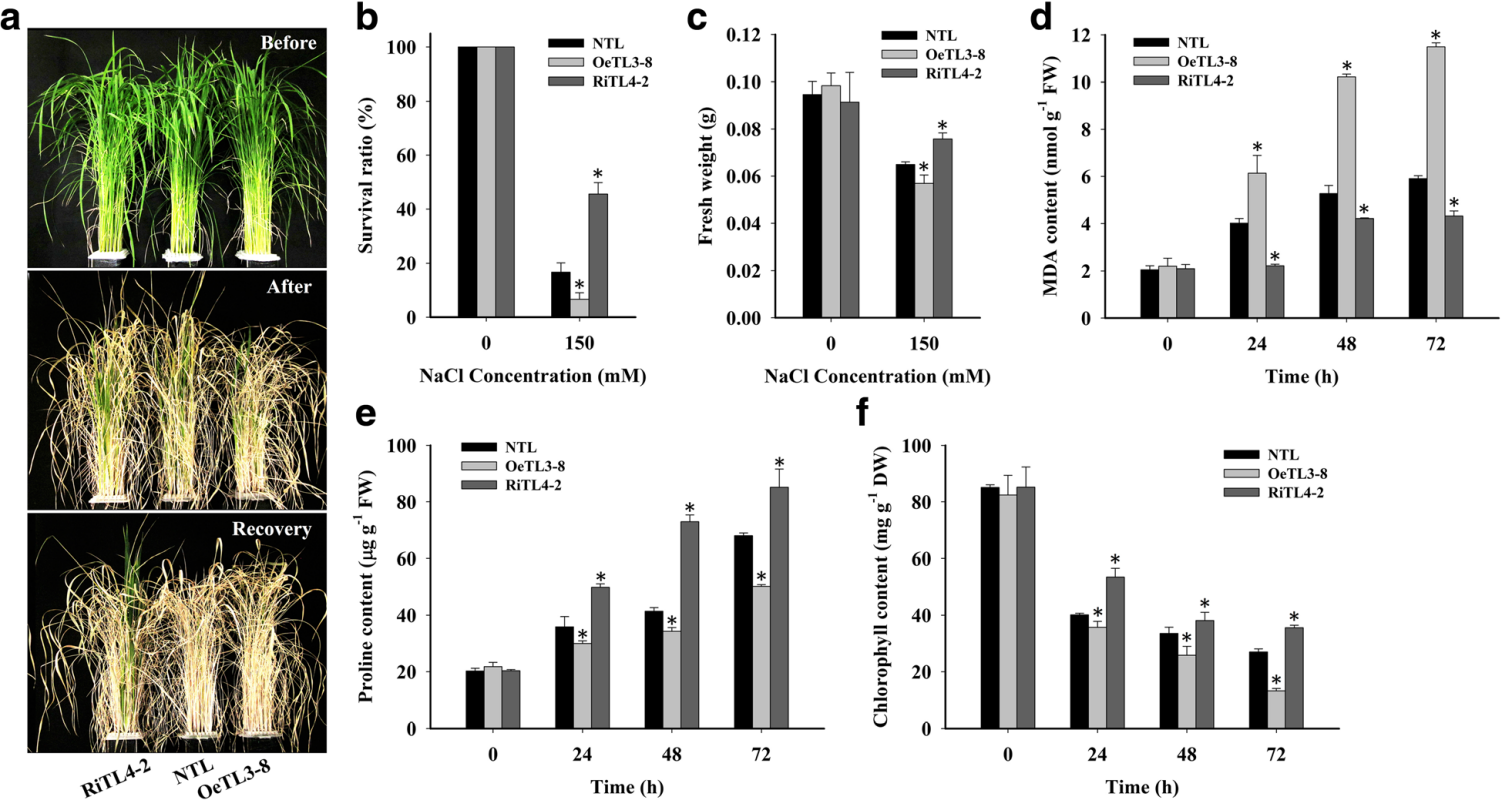
*OsRACK1A* negatively regulates salt tolerance. **a** Comparison of the non-transgenic line (NTL), the *OsRACK1A*-overexpressing transgenic line (OeTL3–8), and the RNA-interfered transgenic line (RiTLs4–2) under 150 mM NaCl for 18 d (salt-stress recovery for 10 d). **b** Survival rate of plants after 150 mM NaCl treatment and recovery. **c** Fresh weight of plants after 150 mM NaCl treatment. **d** to **f** Contents of proline, MDA, and chlorophyll under 150 mM NaCl for 0, 24, 48 and 72 h. Sixty seedlings per genotype (twenty seedlings for each biological replicate) were used. Data shown are the means ± SE of three biological replicates. An asterisk indicates a significant difference (*P* < 0.05) versus stressed NTL

Because both mRNA and protein levels of *OsRACK1A* were induced by the high-salinity treatment, we used the *OsRACK1A*-overexpressing (OeTL3–8) and RNAi (RiTL4–2) lines to determine whether these different transgenic lines showed differences in performance under salt stress versus the NTL. Under normal conditions, transgenic plants showed no significant difference in growth versus the NTL. When 4-week-old plants were stressed with 150 mM NaCl for 18 d, the RiTL4–2 plants had more green leaves than the OeTL3–8 or NTL plants. After 18 d of high-salt treatment, all plants were subjected to normal irrigation (without salt stress) to allow recovery. Only RiTL4–2 plants survived and resumed growth, forming new tillers, while OeTL3–8 and NTL plants died during the 10-d recovery period (Fig. 3a). Twenty pots of plants were counted and the data showed that the survival rate of RiTL4–2 plants was ~ 50%, whereas only ~ 20% of NTL plants survived. The lowest survival rate (< 10%) was observed in OeTL3–8 plants (Fig. 3b). After 10 d of high-salinity treatment, the fresh weight of NTL was significantly higher than that of OeTL3–8 and lower than that of RiTL4–2 (Fig. 3c). These results supported the notion that *OsRACK1A* increases the salt stress response in rice.

To evaluate the effects of salt stress on cell membranes, 4-week-old seedlings were treated with 150 mM NaCl for 24, 48, or 72 h and the malondialdehyde (MDA) content was measured. The RiTL4–2 plants had lower MDA contents, whereas OeTL3–8 contained more MDA than NTL under salt stress (Fig. 3d). The MDA contents indicated that cell membrane stability was reduced in the *OsRACK1A*-overexpressing line and increased in the *OsRACK1A*-RNAi line, versus the NTL under high-salinity stress. Most plants showed increased proline contents under salt-stress conditions, which was considered to be correlated with their stress resistance. In this study, the content of proline was increased after salt stress in plants. Compared with NTL plants, RiTL4–2 plants accumulated higher levels of proline and OeTL3–8 accumulated lower levels under 150 mM NaCl treatment (Fig. 3e). It is known that salt stress causes chlorophyll degradation. We examined the chlorophyll content of rice plants exposed to 150 mM NaCl. As shown in Fig. 3f, the chlorophyll content declined after salt stress. Compared with NTL plants, chlorophyll contents in RiTL4–2 plants were higher, whereas those in OeTL3–8 were lower under 150 mM NaCl treatment. These results indicated that suppression of *OsRACK1A* enhanced salt-stress tolerance.

### *OsRACK1A* regulates Na^+^ and K^+^ levels under salt stress

**Fig. 4:**
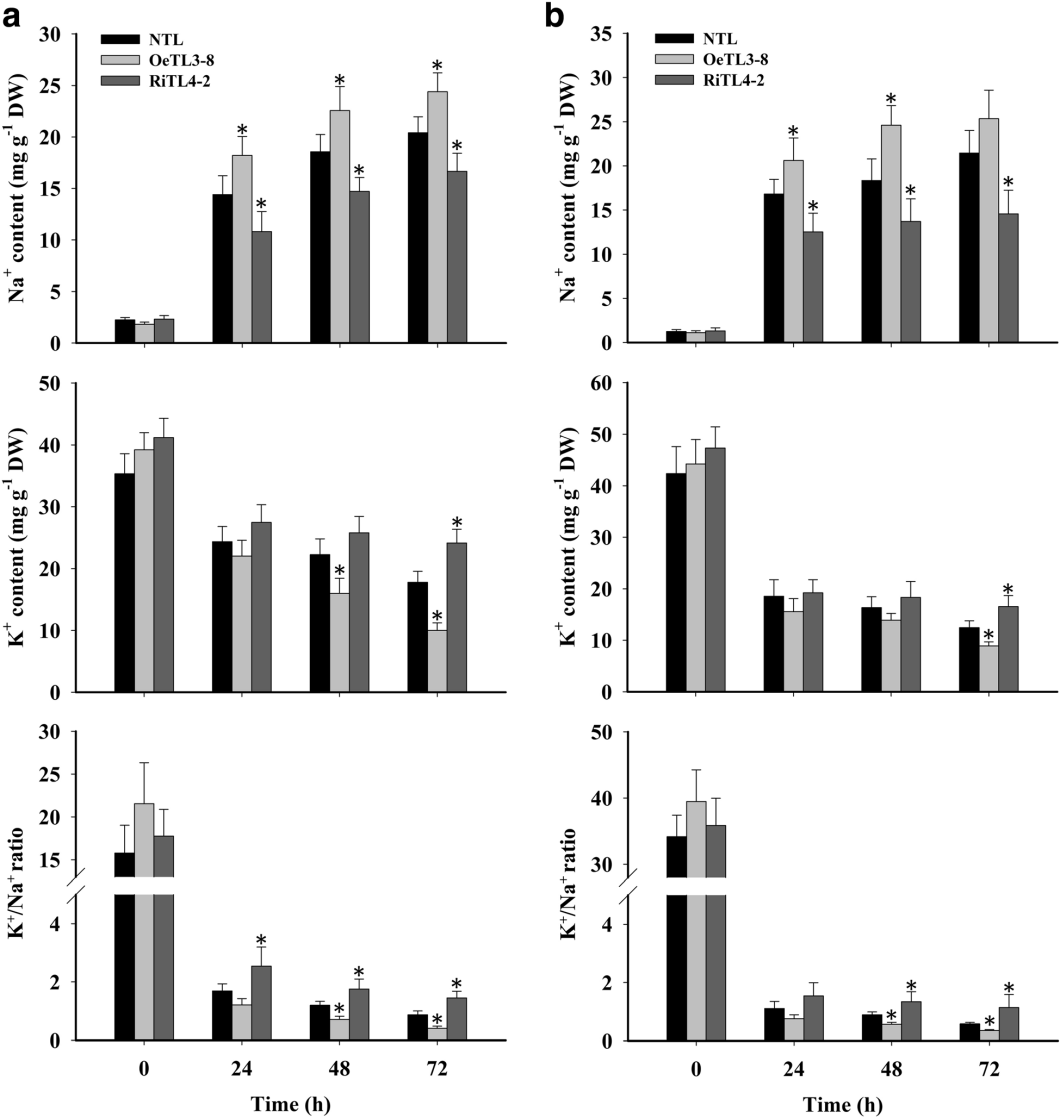
Na^+^ and K^+^ contents in shoots and roots of various rice plants under salt stress. Ion content in leaf (**a**) and root tissue (**b**) of four-week-old NTL, OeTL3–8 and RiTL4–2 plants exposed to salt stress (150 mM NaCl) for 72 h. Values shown are for Na^+^ and K^+^ together with changes in the K^+^/Na^+^ ratio over time. Sixty seedlings per genotype (twenty seedlings for each biological replicate) were used. Data shown are the means ± SE of three biological replicates. An asterisk indicates a significant difference (*P* < 0.05) versus stressed NTL

An important aspect of salt tolerance is the avoidance of Na^+^ accumulation, and K^+^ homeostasis is important for this process (Zhu, 2003). Four-week-old hydroponically grown transgenic and non-transgenic rice plants were subjected to 150 mM NaCl for 72 h. Subsequently, the leaves and roots were harvested at 0 h (before stress) and after 24, 48, and 72 h of salt stress, to measure Na^+^ and K^+^ contents. Before the NaCl treatment, Na^+^ and K^+^ levels in both shoots and roots of the plants with the three different genotypes were similar. The level of Na^+^ increased continuously under salt stress in all plant lines tested. However, RiTL4–2 plants accumulated significantly less Na^+^ than the NTL, whereas OeTL3–8 contained more Na^+^ in both leaves and roots (Fig. 4a). In contrast to the Na^+^ levels, the K^+^ levels declined in the leaves and roots of all plant lines tested during salt stress. At 72 h of salt stress, the content of K^+^ in RiTL4–2 plants was higher than in NTL, whereas OeTL3–8 contained less K^+^ than NTL in leaves and roots (Fig. 4b). These results suggested that RiTL4–2 plants might have the ability to avoid Na^+^ accumulation and maintain K^+^ homeostasis under high-salinity stress. It is generally accepted that the ability to maintain a high K^+^/Na^+^ ratio contributes to salt tolerance in plants (Zhu, 2003). A decreasing K^+^/Na^+^ ratio was detected in the roots and leaves of all plants under salt stress. However, a markedly higher K^+^/Na^+^ ratio was observed in both shoots and roots of the RiTL4–2 plants than those of the NTL and OeTL3–8 plants, whereas no significant difference was observed under normal conditions (Fig. 4a, b). These results imply that *OsRACK1A* negatively regulates rice tolerance of NaCl largely by controlling the Na^+^ and K^+^ accumulation in cells.

### *OsRACK1A* regulates endogenous ABA content and ABA-responsive genes under salt stress

**Fig. 5:**
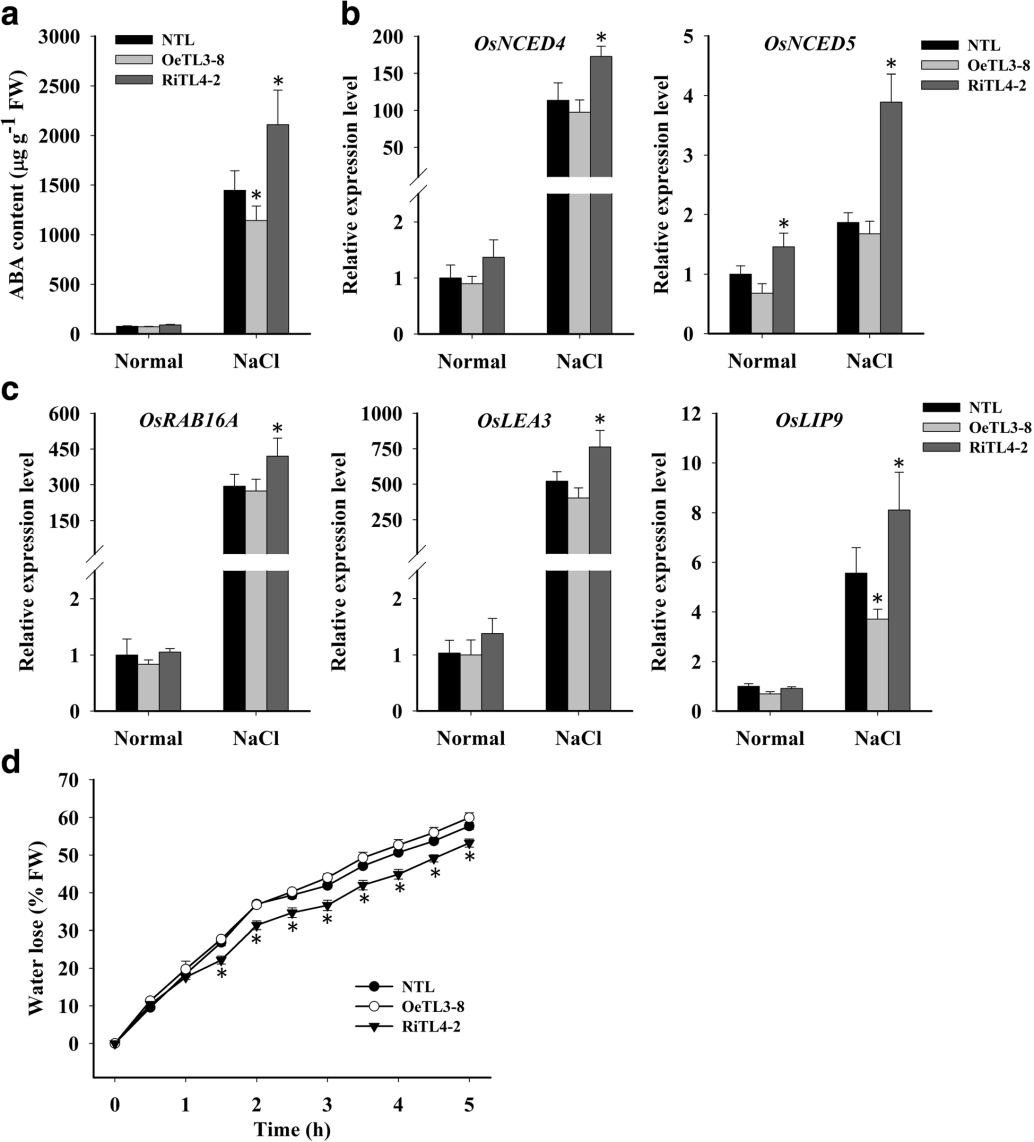
*OsRACK1A* regulates ABA content and ABA-responsive genes under salt stress. **a** ABA content of various rice plants under normal and salt stress (150 mM NaCl). **b** Transcript level of ABA biosynthesis genes, *OsNCED4* and *OsNCED5* in various rice plants under normal and salt stress (150 mM NaCl for 24 h). **c** Transcript level of ABA- and stress-inducible genes in various rice plants under normal conditions and salt stress (150 mM NaCl for 24 h). **d** Water loss in various rice plants. Whole leaves of 14-d-old plants were cut off and used for the water loss assays. Sixty seedlings per genotype (twenty seedlings for each biological replicate) were used. Data shown are the means ± SE of three biological replicates. An asterisk indicates a significant difference (*P* < 0.05) versus stressed NTL

The phytohormone ABA is a crucial regulator of plant growth and development, and plays a critical role in controlling adaptive plant responses to environmental stresses, such as drought, high salt stress, cold stress, and pathogen infection (Cutler et al., 2010; Umezawa et al., 2010). ABA accumulation and some ABA biosynthesis genes are upregulated by NaCl, drought, and cold stress (Cutler et al., 2010). We determined the endogenous ABA content of leaves under salt stress and found that the endogenous ABA content was significantly lower in OeTL3–8, and significantly higher in RiTL4–2 compared with that of NTL (Fig. 5a). The ABA content induced under stress conditions is regulated by the ABA biosynthesis 9-cis-epoxycarotenoid dioxygenase (*NCED*) genes (Xiong et al., 2002). Our preliminary analysis showed that the transcript level of the *OsNCED4* and *OsNCED5* genes was dramatically induced under salt stress (Additional file 1: Figure S2). This study showed the transcript level of *OsNCED4* in RiTL4–3 was ~ 1.5-fold those in NTL and OeTL3–8, and the transcript level of *OsNCED5* was much higher in RiTL4–3 than in NTL and OeTL3–8 under stress conditions (Fig. 5b).

Next, we determined the transcript expression of three ABA response genes– *OsRAB16A*, *OsLEA3* and *OsLIP9* – under salt stress. As shown in Fig. 5c, without NaCl treatment, the transcript levels of *OsRAB16A*, *OsLEA3* and *OsLIP9* showed no significant difference between wild-type and transgenic plants. Upon 150 mM NaCl treatment, transcripts of these genes accumulated significantly in all three genotypes, while RiTL4–3 accumulated more transcripts than NTL and OeTL3–8 in response to salt stress (Fig. 5c). Because ABA is a key regulator of stomatal opening and closure, water loss from the detached leaves of NTL, OeTL3–8 and RiTL4–2 was compared. As shown in Fig. 5d, water loss in RiTL4–2 was much slower than in NTL and OeTL3–8. These results suggested that *OsRACK1A* negatively regulated the expression of ABA-dependent stress-inducible genes under salt treatment conditions.

### *OsRACK1A* significantly changes expression of salt stress-related genes in rice plants

**Fig. 6:**
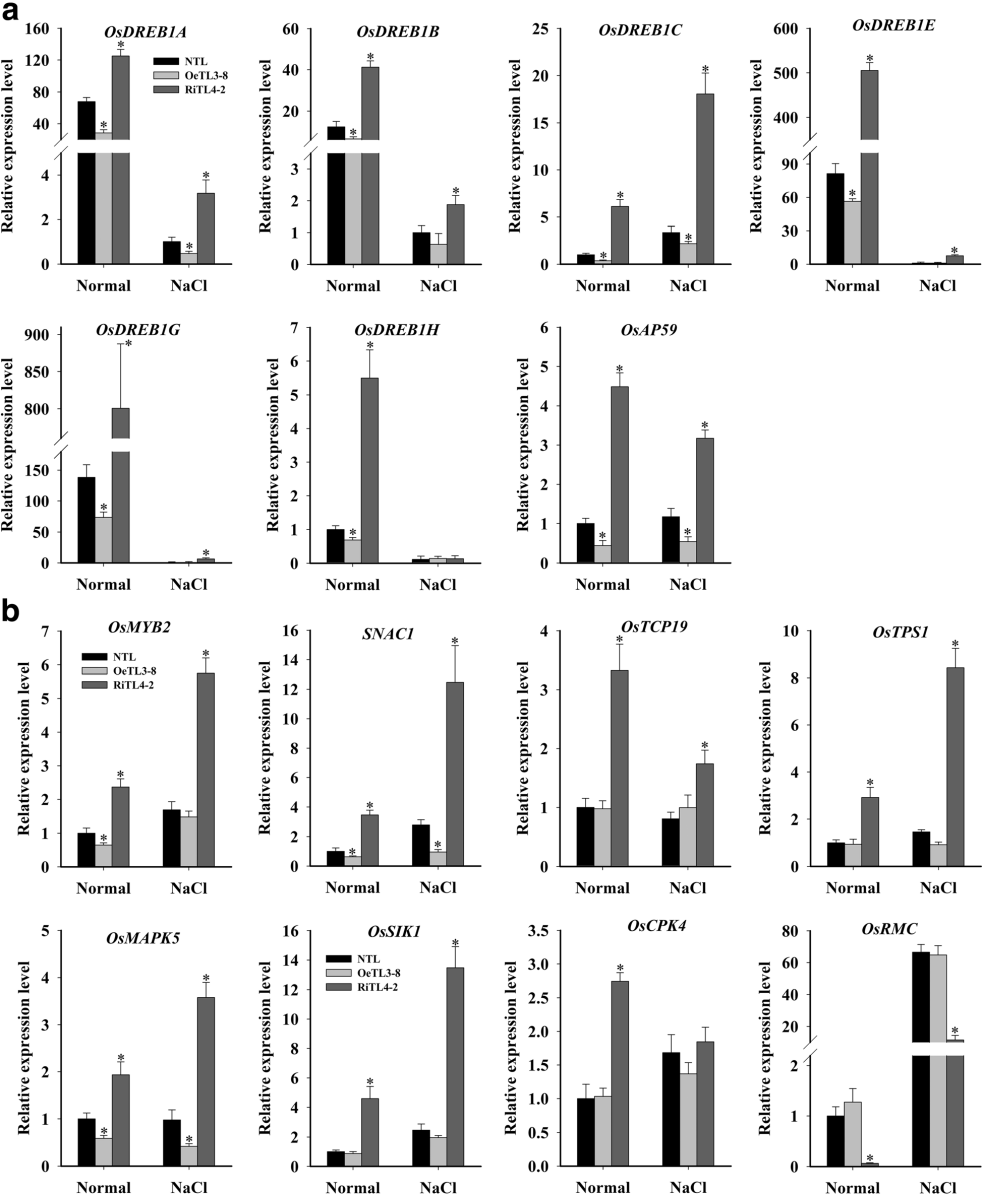
*OsRACK1A* regulates the expression of stress-related genes. Expression of stress-related AP2/ERF genes (**a**) and other stress-related genes (**b**) in OeTL3–8 and RiTL4–2 compared with the NTL in both normal and salt-stress (150 mM NaCl for 24 h) conditions by qRT-PCR. Data shown are the means ± SE of four biological replicates. An asterisk indicates a significant difference (*P* < 0.05) versus stressed NTL

Next, we evaluated the expression of stress-related genes in NTL, OeTL3–8, and RiTL4–2 plants grown under both control and salt-stress conditions by real-time qPCR. As shown in Fig. 6a, under control conditions, the dehydration-responsive element-binding protein 1 (DREB1) genes, *OsDREB-A, -1B*, *-1C*, *−1E*, -*1G*and *-1H*, and the stress-related APETALA2/Ethylene Responsive Factor (AP2/ERF) gene AP59 were upregulated in RiTL4–2 and downregulated in OeTL3–8, in comparison with NTL. When treated with 150 mM NaCl for 24 h, the transcript levels of *OsDREB1A, 1B*, *-1C*, *−1E*, *−1G*, and *OsAP59* in RiTL4–2 were higher than those in NTL, and the expression of OsDREB1A, -1C and OsAP59 was lower in OeTL3–8 (Fig. 6a).

We selected another seven salt stress-responsive genes (*OsMYB2*, *SNAC1*, *OsTCP19*, *OsTPS1*, *OsMAPK5*, *OsSIK1*, and *OsCPK4*) that have been reported to improve salt-stress tolerance (Xiong and Yang, 2003; Hu et al., 2006; Ouyang et al., 2010; Li et al., 2011; Yang et al., 2012; Campo et al., 2014; Mukhopadhyay and Tyagi, 2015). The expression levels of these genes were all upregulated significantly in RiTL4–2, while the expression levels of *OsMAPK5, OsMYB2* and *SNAC1* were downregulated significantly in OeTL3–8. *OsRMC* has been reported to be a negative regulator of the salt-stress response in rice (Zhang et al., 2009; Serra et al., 2013) and the expression of *OsRMC* was downregulated in RiTL4–2 (Fig. 6b). Similar expression profile of these genes occurred under salt-stress conditions (Fig. 6b). These results may partially explain the phenotype of RiTL4–2 plants under stress conditions.

### OsRACK1A interacts with salt-stress response proteins

**Fig. 7:**
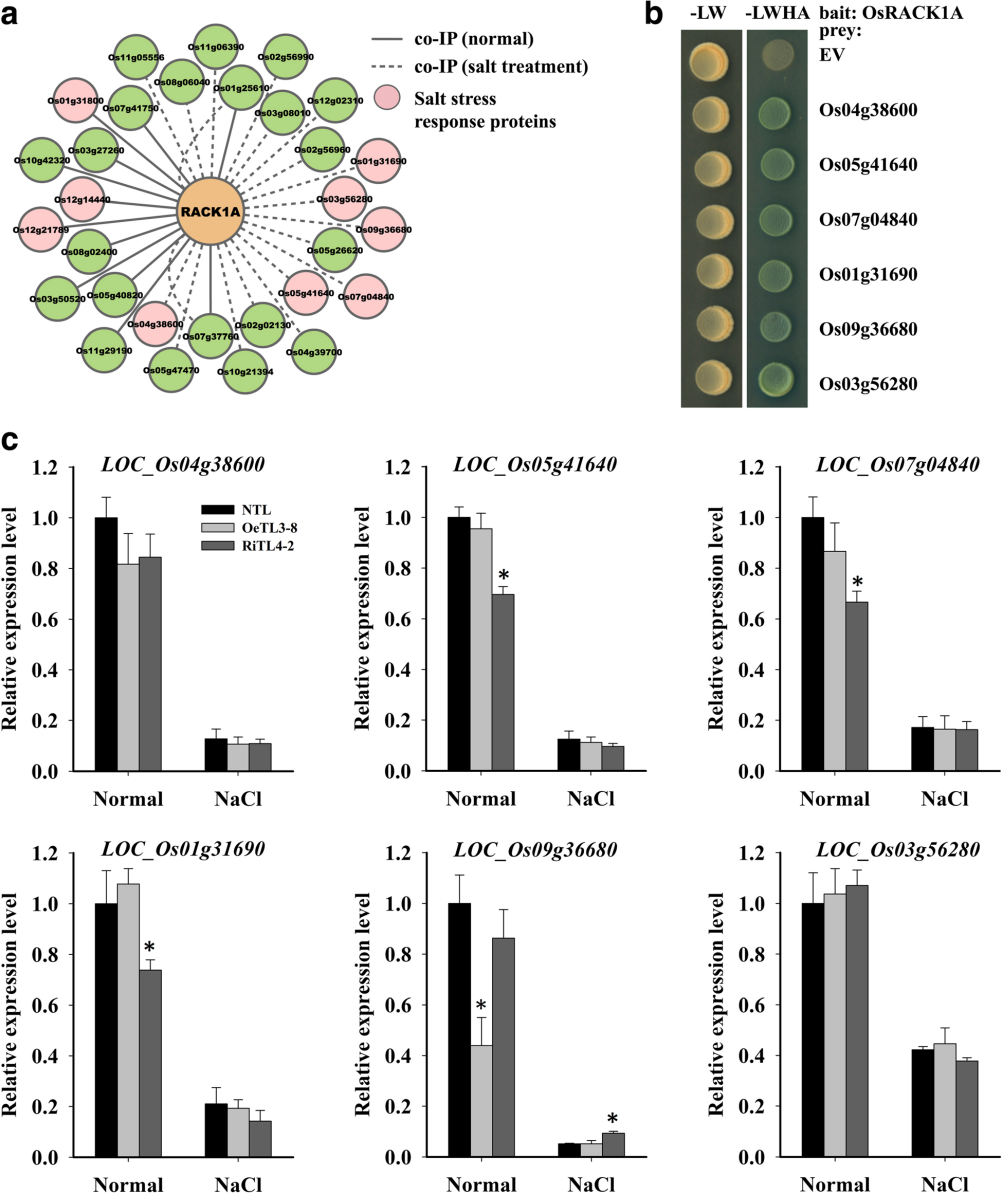
OsRACK1A directly interacts with stress-response proteins. **a** OsRACK1A interaction proteins screening using co-IP in both normal and salt stress (150 mM NaCl for 24 h) conditions. **b** Verifying the protein interactions by yeast two hybrid assay. **c** Quantitative RT-PCR analysis of the expression of OsRACK1A interactors in response to salt stress (150 mM NaCl for 24 h). Data shown are the means ± SE of four biological replicates. An asterisk indicates a significant difference (*P* < 0.05) versus stressed NTL

As a scaffold protein, RACK1 interacts with numerous proteins and plays a critical role in many fundamental physiological processes, including stress responses (Zhang et al., 2013). In this study, we used co-immunoprecipitation (co-IP) to identify novel proteins that interact with OsRACK1A under both normal and salt-stress conditions. As shown in Fig. 7a and Additional file 2: Table S1, 12 and 20 proteins were detected to interact with OsRACK1A directly or indirectly in normal and stress conditions, respectively. Of these 32 identified proteins, two (Os07g37760 and Os01g25610) interacted with OsRACK1A in both normal and salt-stress conditions (Fig. 7a). Nine of these genes responded to NaCl treatment (Additional file 1: Figure S3). We also used the yeast two-hybrid assay to confirm these interactions and ultimately found that six of the identified proteins interacted directly with OsRACK1A (Fig. 7b). Interestingly, these six proteins were all identified in salt-stress conditions by co-IP assay and their mRNA levels were all downregulated with salt treatment (Fig. 7a, Additional file 1: Figure S3). The mRNA expression of these six OsRACK1A interacting proteins was also detected in NTL, OeTL3–8, and RiTL4–2 under both normal and salt-stress conditions. The transcript levels of Os05g41640, Os07g04840, and Os01g31690, which encode phosphoglycerate kinase, PsbP, and oxygen-evolving enhancer protein 1, respectively, were lower in RiTL4–2 compared with NTL and OeTL3–8 in normal conditions, whereas no significant change in the expression of these genes was observed in NTL and transgenic plants after NaCl treatment (Fig. 7c). The Os09g36680, which encodes a ribonuclease T2 family domain containing protein, was downregulated in OeTL3–8 with no treatment and up-regulated in RiTL4–2 under salt treatment (Fig. 7c). These results indicated that OsRACK1A regulates stress responses by interacting directly with many stress related proteins.

## Discussion

RACK1 is a highly conserved scaffold protein that is expressed ubiquitously (Zhang et al., 2013). *RACK1* is involved in multiple signaling pathways, including growth and development and responses to external environmental stresses (McCahill et al., 2002; Zhang et al., 2013). However, the molecular mechanisms of RACK1 in plants is still in its infancy. In plant, *RACK1* is involved in the regulation of cell proliferation and elongation, and the responses to plant hormones and environmental factors (Chen et al., 2006; Nakashima et al., 2008; Guo et al., 2009; Li et al., 2009; Zhang et al., 2013; Zhang et al., 2014). The rice genome contains two *RACK1* ortholog genes, *OsRACK1A* and *OsRACK1B* (Nakashima et al., 2008). Although *OsRACK1A* and *OsRACK1B* are similar, *OsRACK1A* transcript levels are always significantly higher than those of *OsRACK1B* in leaves, roots, and mature seeds (Zhang et al., 2014). Previously, we reported that *OsRACK1A* negatively regulated the response of seed germination to exogenous ABA and the suppression of *OsRACK1A* improved drought tolerant in rice (Li et al., 2009; Zhang et al., 2014). In the present study, we showed that *OsRACK1A* negatively regulated salt stress tolerance and sought to explore the molecular mechanism(s) involved.

A circadian oscillator controls the timing of several physiological functions in living organisms. In plants, processes controlled by a circadian clock include the photoperiodic induction of flowering, rhythmic leaf movements and stomatal opening (Thines and Harmon, 2011). Recent research also suggests that a circadian clock may contribute to plant fitness, enhancing their ability to tolerate abiotic stress (Grundy et al., 2015). In maize, transcripts of many stress related genes exhibits a diurnal cycling pattern (Hayes et al., 2010; Khan et al., 2010). Some salt stress-responsive genes, such as *SOS1*, *RD29A* and *DREB2A*, exhibit a 24-h period of expression in *Arabidopsis*, suggesting that salt tolerance may also be affected by the circadian clock (Park et al., 2016a). In some cases, other stress, such as cold and drought, modifies the transcription pattern of a major portion of genes showing diurnal oscillation (Wilkins et al., 2010; Jończyk et al., 2017). Resent evidence indicates that plants respond to salt stress more strongly during the day than at night and salt-induced expression of RD29A and SOS1 was much higher in the daytime than at night (Park et al., 2016b). We found that both mRNA and protein levels of *OsRACK1A* exhibits a diurnal cycling pattern, and much higher during the day than at night (Fig. 1a, b). However, expression of OsRACK1A increased under salt stress and remained high in both the light and dark (Fig. 2a, b). It might be that higher expression levels of OsRACK1A in day caused more damage under salt stress.

In rice, OsRACK1A protein is phosphorylated under ABA and drought treatment, although the kinase responsible was not identified (He and Li, 2008; Ke et al., 2009). Recently, Urano et al. (2015) showed that *Arabidopsis* RACK1A (AtRACK1A) is also phosphorylated by an atypical serine/threonine protein kinase, WITH NO LYSINE 8 (WNK8), and phosphorylation of AtRACK1A rendered it unstable. Interestingly, in this present study, we found that OsRACK1A protein was controlled by post-transcriptional or translational regulation and consequently accumulated under salt stress. These results led us to the hypothesize that phosphorylation of OsRACK1A does not reduce the protein stability in rice and RACK1 protein may play distinct roles in different plant species. Guo and Sun (2017) found that sumoylation of *Arabidopsis* RACK1B (AtRACK1B) increased AtRACK1B stability and its tolerance to ubiquitin-mediated degradation in the ABA response, and consequently enhanced the interaction between RACK1B and RAP2.6. Combined, these findings illustrate that protein stability controlled by post-transcriptional modification is a critical regulatory mechanism for RACK1 in both *Arabidopsis* and rice.

In *Arabidopsis*, the clock component GIGANTEA (GI) is involved in salt-stress responses (Kim et al., 2013). Similar to *OsRACK1A*, *GI* transcription is under circadian control and peaked at 8–10 h after the start of the day (Park et al., 1999). Under normal conditions, GI interacts with SOS2, a key component of the SOS pathway, preventing the interaction between SOS2 and SOS3. Under salt stress conditions, GI is degraded and the free SOS2/SOS3 complex activates SOS1, a Na^+^/H^+^ antiporter, to export sodium (Na^+^) ions from cells (Kim et al., 2013). In the present study*, OsRACK1A* also negatively regulated Na^+^ accumulation and subsequently maintained a low K^+^/Na^+^ ratio in rice seedlings under NaCl stress (Fig. 4a, b). We investigated the proteins that interact with OsRACK1A and identified six salt-stress suppressed proteins that interacted with OsRACK1A directly (Fig. 7a, b). Unfortunately, none of these proteins were reported to be directly involved in salt-stress responses and the relationship between *OsRACK1A* and the Na^+^/H^+^ antiporter is still unclear. In eukaryotes, RACK1 regulates various signaling pathways and cellular processes through its interaction with numerous signaling proteins (Zhang et al., 2013). For example, OsRACK1A binds the active form of Rac1 and interacts with the N terminus of Rboh, RAR1, and SGT1, to form a complex in rice innate immunity (Nakashima et al., 2008). Similarly, OsRACK1A may form a complex with these salt-stress responses proteins, and active downstream molecules, such as salt-stress relative transcription factor. Future studies will reveal whether these OsRACK1A-interaction proteins are involved in salt-stress response.

Under high-salt-stress conditions, a key plant stress-signaling hormone, ABA, and numerous ABA-induced stress-responsive genes products accumulate (Yoshida et al., 2014). The *NCED* genes are known to encode key enzymes in ABA biosynthesis in plants (Nambara and Marion-Poll, 2005). In *Arabidopsis*, *AtNCED3* is induced by drought and high salinity, and the overexpression of *AtNCED3* in transgenic plants enhanced dehydration stress tolerance (Iuchi et al., 2001). Five *NCED* genes (*OsNCED1–5*) have been identified in the rice genome (Zhu et al., 2009). The qPCR analysis showed that the *OsNCED4* and *OsNCED5* were induced strongly under salt stress (Additional file 1: Figure S2), suggesting that transcriptional regulation of the *OsNCED4* and *OsNCED5* genes may be involved in salt-induced ABA accumulation in rice. Previous study has showed that *OsRACK1A* negatively regulated the response of seed germination to exogenous ABA (Zhang et al., 2014). Here we showed that the expression levels of *OsNCED4* and *OsNCED5* were higher in the *OsRACK1A*-suppressed line (RiTL4–2) than the non-transgenic line and the *OsRACK1A*-expressing line (OeTL3–8; Fig. 5b). Additionally, the ABA content was higher in RiTL4–2 than in NTL and OeTL3–8 (Fig. 5a), suggesting that the OsRACK1A protein suppressed ABA accumulation under salt stress by regulating the expression of ABA biosynthesis genes. Some typical ABA-dependent stress-inducible genes, such as *OsRAB16A*, *OsLEA3* and *OsLIP9*, show higher mRNA levels in RiTL4–2, indicating that *OsRACK1A* is involved in ABA-dependent stress pathways.

The AP2/ERF transcription factor superfamily is involved in responses to biotic and abiotic stresses, the regulation of metabolism, and developmental processes in various plant species (Dossa et al., 2016). We selected some *AP2/ERF* genes (*OsDREB-1A*, *1B*, *-1C*, *−1E*, *−1G*, *-1H* and *OsAP59*) and found that these *AP2/ERF* genes were all upregulated in RiTL4–2 and some of them were downregulated in OeTL3–8 (Fig. 6a). Many of the upregulated *AP2/ERF* genes have been reported to play roles in salt-stress tolerance. Transgenic plants overexpressing *OsDREB1B* showed higher tolerances to drought, high salt, and freezing stresses (Dubouzet et al., 2003; Qin et al., 2006; Mao and Chen, 2012). The *OsAP59* gene was found to be induced after exposure to drought and high-salt conditions, and constitutive expression of *OsAP59* in rice increased the tolerance to drought and high salinity during vegetative development (Oh et al., 2009). Some of these *AP2/ERF* genes, such as *OsAP59*, were not induced by ABA (Oh et al., 2009). These results suggested that *OsRACK1A* is also involved in ABA-independent signaling in response to stress in rice. We found some other stress-related transcription factors, such as *OsMYB2*, *SNAC1* and *OsTCP19* were upregulated in *OsRACK1A* suppressed-expression plants in both normal and stress condition (Fig. 6b). OsMYB2-overexpressing plants were reported showing more tolerant to salt, cold, and dehydration stresses and more sensitive to abscisic acid than wild-type plants (Yang et al., 2012). Interestingly, two core circadian clock components, CIRCADIAN CLOCK ASSOCIATED 1 (CCA1) and LATE ELONGATED HYPOCOTYL 1 (LHY1), are also belone to MYB transcription factor family and involved in cold stress responses (Dong et al., 2011), suggesting that MYB transcription factors might be molecular link between circadian clock and stress responses. We also showed that suppression of *OsRACK1A* activated several known stress-related kinases, such as *OsSIK1*, *OsMAPK5*, and *OsCPK4* (Fig. 6b). These genes have been reported to be induced by cold, drought, salinity, ABA, and other abiotic stresses. Transgenic plants overexpressing these genes exhibited enhanced tolerance to various stresses (Xiong and Yang, 2003; Ouyang et al., 2010; Campo et al., 2014). In addition, we showed that expression of *OsRMC*, which negatively regulates salt-stress tolerance in rice (Serra et al., 2013), was suppressed in the RiTL4–2 line (Fig. 6b). Although the signal transduction pathway involving these gene products is unclear, we suggest that *OsRACK1A* participates in abiotic stress pathways, directly or indirectly, by altering the expression of these stress-related genes.

## Conclusions

In summary, results presented in this study demonstrate that OsRACK1A functions as a stress-responsive gene and *OsRACK1A* RNAi transgenic rice can significantly improve salt stress tolerance through ABA-dependent and -independent pathway. As a negative regulator of salt stress response, *OsRACK1A* expresses rhythmically under normal conditions and shows the loss of cycling under salt stress. Although OsRACK1A interacts with many salt-responsive proteins, no directly evidence links OsRACK1A protein to salt stress related transcription factors, such as DREB and AP2/ERF. Further investigations on the identification of the functions of OsRACK1A interaction proteins will be helpful to elucidate the mechanism of *OsRACK1A* in regulating salt stress tolerance.

## Additional files

Additional file 1: **Table S2.** Primers used for the qRT-PCR analysis of various genes. **Figure S1.** Public microarray data showing *OsRACK1A* (A) and *OsRACK1B* (B) expression is controlled by a circadian clock (http://www.ebi.ac.uk/arrayexpress/experiments/E-MTAB-275/). *OsRACK1A* expression in rice leaves under 16 h light/ 8 h dark (LD) or constant light (LL) conditions and under NaCl treatment (C). **Figure S2.** *OsNCED* gene expression in rice leaves under 150 mM NaCl treatment for 12 h. **Figure S3.** Quantitative RT-PCR analysis of the expression of OsRACK1 interactors in response to salt stress. (DOCX 356 kb)

Additional file 2: **Table S1.** Identification of OsRACK1A interacting proteins. (XLSX 22 kb)

## Abbreviations

ABA: Abscisic acid
AP2/ERF: Apetala2/ethylene responsive factor
BMAL1: Brain and muscle arnt-like protein-1
BR: Brassinosteroid
DREB: Dehydration responsive element binding protein
ERFs: Ethylene response factors
GA: Gibberellin
H_2_O_2_: Hydrogen peroxide
IAA: Indole-3-acetic acid
MDA: Malondialdehyde
NCED: 9-cis-epoxycarotenoid dioxygenase
NTL: Non-transgenic line
OeTL: Overexpressing-transgenic line
qPCR: Quantitative PCR
RACK1: Receptor for activated C kinase 1
RiTL: RNA interference- transgenic line
ROS: Reactive oxygen species
SOS: salt overly sensitive
WD40: Tryptophan-aspartic acid 40
ZT: Zeitgeber time
