# Supplementary material for: OsRACK1A, encodes a circadian clock-regulated WD40 protein, negatively affect salt tolerance in rice
Source: Rice (N Y). 2018 Aug 2;11:45. doi: 10.1186/s12284-018-0232-3 (PMC6081827; doi:10.1186/s12284-018-0232-3)
Supplement: Supplementary file 1 — Table S2. Primers used for the qRT-PCR analysis of various genes. Figure S1. Public microarray data showing OsRACK1A (A) and OsRACK1B (B) expression is controlled by a circadian clock (http://www.ebi.ac.uk/arrayexpress/experiments/E-MTAB-275/). OsRACK1A expression in rice leaves under 16 h light/ 8 h dark (LD) or constant light (LL) conditions and under NaCl treatment (C). Figure S2. OsNCED gene expression in rice leaves under 150 mM NaCl treatment for 12 h. Figure S3. Quantitative RT-PCR analysis of the expression of OsRACK1 interactors in response to salt stress. (DOCX 356 kb) [file 12284_2018_232_MOESM1_ESM.docx]

**Table S2.** Primers used for the qRT-PCR analysis of various genes

| Gene | Locus name | Primers |
| --- | --- | --- |
| *OsActin1* | LOC_Os03g50890 | F: ACAGGTATTGTGTTGGACTCTGG  R: AGTAACCACGCTCCGTCAGG |
| *OsRACK1A* | LOC_Os01g49290 | F: AGGGATCTGTTTTGCACCAT  R: GAGAGAAGCACCATGGATCG |
| *OsRAB16A* | LOC_Os11g26790 | F: CATGGACAAGATCAAGGAGAAGC  R: CTTATTATTCAGGAAGGTGACGTGG |
| *OsLEA3* | LOC_Os05g46480 | F: GCCGTGAATGATTTCCCTTTG  R: CACACCCGTCAGAAATCCTCC |
| *OsLIP9* | LOC_Os02g44870 | F: TGGAATTTGGAAGTGTTTGGC  R: CCCACACGAAACACAAACTTC |
| *OsNCED4*  *OsNCED5*  *OsAP59*  *OsSIK1*  *OsTPS1*  *OsMAPK5*  *SNAC1*  *OsTCP19*  *OsCPK4*  *OsMYB2*  *OsRMC* | LOC_Os07g05940  LOC_Os12g42280  LOC_Os02g43790  LOC_Os06g03970  LOC_Os08g34580  LOC_Os03g17700  LOC_Os03g60080  LOC_Os06g12230  LOC_Os02g03410  LOC_Os03g20090  LOC_Os04g56430 | F: ATCTCCTTCTCCCTCCTCCCA  R: TCGCACCCTGCTTGATCTTGC  F: TCCGAGCTCCTCGTCGTGAA  R: AGGTGTTTTGGAATGAACCA  F: GGTGATTTAGCCATCTTGTGCG  R: TCGTCACATTTCTTGGAGCAG  F: TCTGGTAGTCTGCCCGAGGAA  R: TATGTACTGGTTGCAATCAG  F: ACCAAGCATACCGCCAGAC  R: ACCATTCAGCCAGTTCATCCT  F: CGACTTCGAGCAGAAGGCTCTA  R: GTTCATCTCGATCGCTTCGTT  F: TGGGAGAAGATGCAGCAGG  R: GAGTGCGACTGCGACGTAAC  F: TTTTCTCCGTTTTTGTTTGAGTTG  R: CATGAATATATGATGGGTCGAGGAA  F: CGTGTGCAGCATGCAGATAA  R: TGCGATGAATACGTGCAATCA  F: GGGCTGAAACGCACAGGCAAGA  R: CTGCTTGGCGTGCTTCTGC  F: TCGGAGGTGTACCCGTTCTACA  R: ACTCTTAATTTGTGCCATTTTATTCTA |

**Table S2.** Continued.

| Gene | Locus name | Primers |
| --- | --- | --- |
| *OsDREB1A*  *OsDREB1B* | LOC_Os09g35030  LOC_Os09g35010 | F: CCACACTCGAGCAGAGCAAAT  R: GCTTGATCCCGCACATCTTC  F: TCTCCGGCGGAGACCTTC  R: CCGGCAACACGTCCTTGT |
| *OsDREB1C* | LOC_Os06g03670 | F: CAAAGCTTATCAGCAGTAGC  R: GGTTAGTAGCAGAAAGACTTG |
| *OsDREB1E* | LOC_Os04g48350 | F: GAATTCGAAATGCAGGGGTA  R: CTCGCAGTCGTAGTCCTCCT |
| *OsDREB1G* | LOC_Os02g45450 | F: CCCGTACTACGAGGTCATGG  R: GCTACCTACGGCAGGATCAC |
| *OsDREB1H*  *OsDREB2A* | LOC_Os09g35020  LOC_Os01g07120 | F: TGCCTCAACTTCCAGGACTC  R: GCCGTTCGTGGTCTTGTTG  F: GGAATCTCCTCCTTTCATCGTG  R: TTCCGCTCCTGACAAACACG |


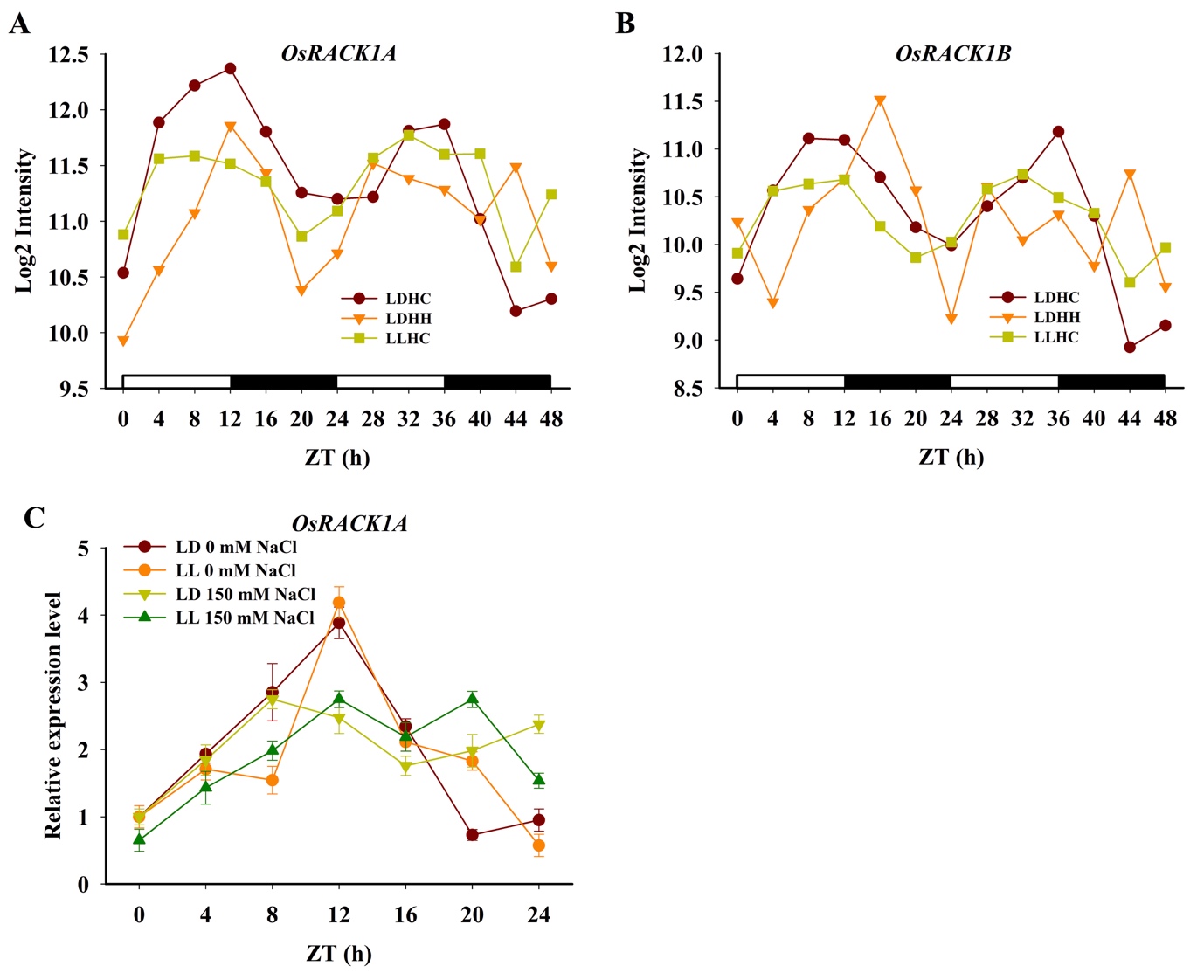


**Figure S1.** Public microarray data showing *OsRACK1A* (A) and *OsRACK1B* (B) expression is controlled by a circadian clock (<http://www.ebi.ac.uk/arrayexpress/experiments/E-MTAB-275/>). *OsRACK1A* expression in rice leaves under 16 h light/ 8 h dark (LD) or constant light (LL) conditions and under NaCl treatment (C).

**
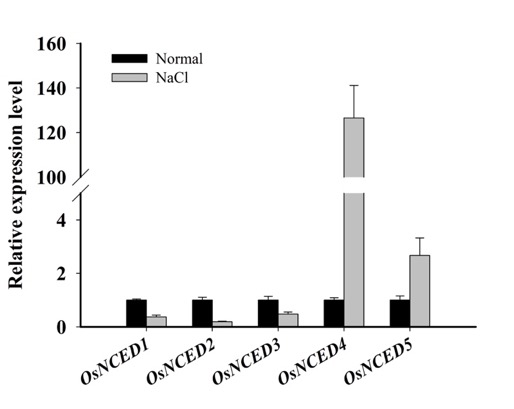
**

**Figure S2.** *OsNCED* gene expression in rice leaves under 150 mM NaCl treatment for 12 h.


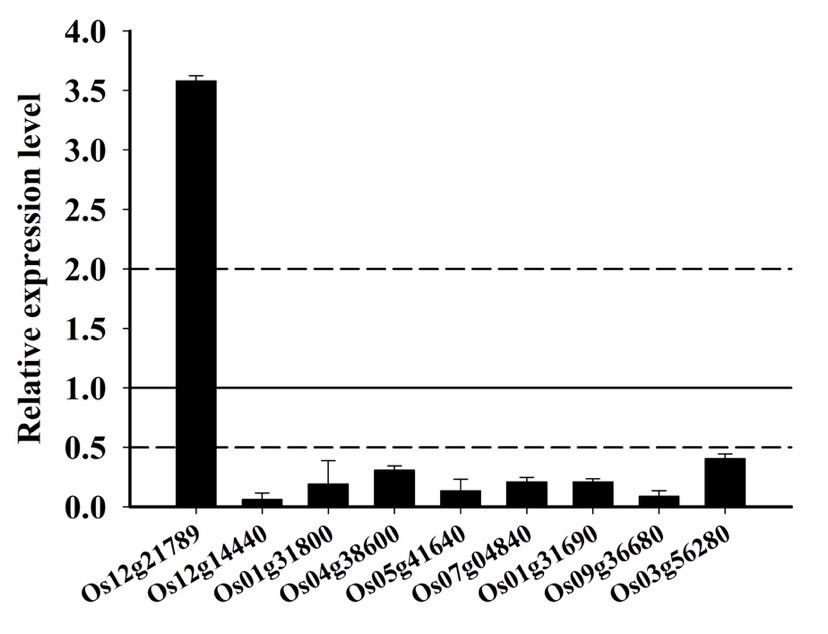


**Figure S3.** Quantitative RT-PCR analysis of the expression of OsRACK1 interactors in response to salt stress.
